# Domestic Correspondence

**Published:** 1889-12

**Authors:** 


### Domestic Correspondence.

---

To the Editor:

After reading the query of Dr. Quick in your journal, and reflecting a moment upon the course pursued by your Society in referring the same to a committee, it is suggested to my mind that any report of cases at all similar might be of service to all concerned.

I would therefore ask the privilege of reporting the following:

Mr. A——, aged about thirty-four years, an Englishman by birth, a minister of the gospel, married, and generally enjoying good health, I learned was confined to his house, and had been compelled to think seriously of resigning his position, and wait patiently for the final summons to eternal rest. By accident his sister became one of my patients, and, while I was operating upon her teeth one day, she happened to mention her brother's condition, and gave me the following history of the case: About four years previous he was attacked with severe neuralgic pains in both temporal regions, together with soreness in the angle of the jaws and great depression of spirits. Accompanying these paroxysms would be several days of extreme trismus and almost starvation, owing to the inability to perform the act of mastication; and as his denture was almost perfect, his attendants had found it difficult to supply him with even liquid nourishment. Periodically this condition of affairs would recur, until, finally, suppuration took place, and though the pus escaped from the corners of his mouth, with its usual concomitant symptoms, his physicians found it impossible to locate the origin of the pyogenetic condition,—and advised him to wait for further developments. The patient was finally advised that his teeth might possibly have something to do with his trouble, and to consult a dentist; this he did, and was told that his teeth were all sound, and he must seek relief from some physician. As stated above, this condition continued for about four years, alternating between the most agonizing pain and some slight occasional improvement, until despair found a habitat in the mind of our friend, and with true Christian resignation he had drawn his mantle around him and laid him down to die.

Inasmuch as his sister told me that he had discarded all professional attendance, I thought I should violate no section of ethics in asking an interview. I did, and was allowed to see the patient, In a little while, I felt convinced that the difficult and almost impossible eruption of the wisdom tooth was the main etiological factor in the pathology of the case. Strange to say, the right lower first molar had been lost, and the third was in sight, though the trouble was as acute on that side as the other, and the effusion as great. I thought, however, this might be sympathetic, and with liniments and massage succeeded in about a week in getting sufficient space between the jaws to admit my forceps, when I extracted the left lower second molar (with great difficulty), and dismissed the patient with an antiseptic mouth-wash. Suffice it to say, recovery has been complete, and the patient is following again his chosen profession, well and happy, though surprised that so much could be accomplished by the extraction of a tooth.

E. L. Clifford, D.D.S.

Chicago, Ill., July 20.

---

To the Editor:

I herewith send you for publication the programme for our January meeting as far as we have gone:

We hold our meetings on January 14, 15, 16, at (Grand Lodge rooms) Masonic Temple, Twenty-third and Sixth Avenue, and the clinic on the 15th and 16th, at the New York College of Dentistry, Second Avenue and Twenty-third Street.

We have three papers written expressly for this meeting,—an illustrated paper ("Photo-micrographs") showing the pathological conditions of the teeth, by Dr. George S. Allan, New York City; a combination paper, by Drs. E. T. Darby and Edward C. Kirk, of Philadelphia; and one by Eugene S. Talbot, of Chicago. These papers will be fully discussed by persons specially invited to do so; and we will remain all night, if necessary, in order to accomplish it. One great fault with previous meetings has been that papers were never fully discussed; but on this occasion they will be.

We will not have dental displays or exhibits of any kind; but, strictly speaking, a dental meeting. I find it a very hard task to get *all dental manufacturing companies* to take part. So rather than have a sort of institute-gain machine-shop and blacksmith display, it has been decided to have no exhibition. H. J. McKellops, M.D., D.D.S., of St. Louis, Mo., has kindly consented to act as supervisor of clinics, in connection with Dr. C. V. Jackson, of New York City.

I also send you the names of those who will clinic WITHOUT FAIL. Dr. T. H. Parramore, Hampton, Va.; Dr. George Enbark, Birmingham, Ala.; Dr. Steward Arnold, Aniston, Ala.; Dr. T. S. Waters, Baltimore, Md.; Dr. Frank B. Darby, Elmira, N. Y.; Dr. F. T. Van Woert, Brooklyn, N. Y.; Dr. H. A. Parr, New York City; Dr. C. S. Stockton, Newark, N. J.; Dr. A. W. Harlan, Chicago, Ill.; Dr. S. C. G. Watkins, Mount Clair, N. J.; Dr. T. C. Shumway, Plymouth, Mass.; Dr. J. Albert Kimball, New York City; Dr. W. R. Clifton, Waco, Tex.; Dr. M. A. Bland, Charlotte, N. C.; Dr. George McElhany, Columbus, Ga.; Dr. George H. Wells, Augusta, Ga.; Dr. George H. Winkler, New York City; Dr. H. H. Sisson, New York City; Dr. J. S. Campbell, London, England; Dr. Wm. H. Dwinelle, New York City; Dr. E. Parmly Brown, New York City; Dr. R. Ottalingue, New York City; Dr. George Evans, New York City; Dr. R. B. Adair, Gainsville, Ga.; Dr. Thomas J. Moore, Columbus, N. C.; Dr. G. L. Curtis, Syracuse, N. Y.; and others that we are expecting to hear from very soon.

W. W. WALKER,
*Chairman Executive Committee.*

---

### RESOLUTIONS ON THE DEATH OF DR. LEON RIDEOUT.

WHEREAS, The Alumni Association of Boston Dental College has been called upon to mourn the loss of one of its members and a past president, Dr. Leon Rideout, of Lynn, Mass.

*Resolved*, That we mourn the loss of our associate, a man of integrity and uprightness, who was ever earnest to elevate the profession and advance its interests; respected in the community in which he lived. May our memory of him be always kept fresh by the good we know he did to mankind.

(Signed) E. O. KINSMAN,

S. G. STEVENS,

J. K. KNIGHT,

} *Committee on Resolutions.*

WM. RICE,

*Secretary.*

BOSTON, 1889.

